# Functional limitations and disability among older adults in Nepal

**DOI:** 10.1093/geroni/igaf122.514

**Published:** 2025-12-31

**Authors:** Usha Dhakal, Hardik Sapkota, Indra Chaudhary, Adina Gurung, Emily Briceño, Uttam Sharma, Dirgha Ghimire, Carlos Mendes de Leon

**Affiliations:** Georgetown University, Washington, District of Columbia, United States; Institute for Social and Environmental Research-Nepal (ISER-N), Chitwan, Bagmati, Nepal; Institute for Social and Environmental Research-Nepal (ISER-N), Chitwan, Bagmati, Nepal; Institute for Social and Environmental Research-Nepal (ISER-N), Chitwan, Bagmati, Nepal; University of Michigan Medical School, Ann Arbor, Michigan, United States; Institute for Social and Environmental Research-Nepal (ISER-N), Chitwan, Bagmati, Nepal; University of Michigan, Ann Arbor, Michigan, United States; Georgetown University, Washington, District of Columbia, United States

## Abstract

The burden of disability among older adults in Nepal remains unclear and understanding this can help inform healthcare services and support systems to enhance their quality of life. Therefore, we examined patterns of disability as a function of functional limitations and other key predictors of late-life disability among older adults in Chitwan, Nepal. Data for this study came from 144 adults 50 years and older using a cluster random sampling design. Nearly one-third (30%) of the participants had at least one activity of daily living (ADL) disability and more than half (52%) had at least one instrumental ADL (IADL) disability. Functional limitations were assessed using physical performance tests of gait speed, chair stand, and grip strength summarized in a composite score (0-9). We used negative binomial regression models to predict the number of ADL and IADL disabilities (0-12) and found that higher physical performance test scores were associated with lower disability after adjusting for age and gender (β=-0.12, p = 0.03). This association was slightly attenuated after adjusting for education and lifetime income (β=-0.10, p = 0.07) and no longer significant after additional adjustment for chronic conditions (β=-0.07, p = 0.19). In separate models for ADL and IADL disability, physical performance tests scores were only predictive of IADL disability. Our findings suggest a substantial burden of disability among older adults in Chitwan, even in this relatively young older age group. We may have lacked sufficient statistical power to more firmly establish the role of functional limitations in the disability process in this population.

